# The risk of developing cancer following metal-on-metal hip replacement compared with non metal-on-metal hip bearings: Findings from a prospective national registry “The National Joint Registry of England, Wales, Northern Ireland and the Isle of Man”

**DOI:** 10.1371/journal.pone.0204356

**Published:** 2018-09-20

**Authors:** Linda P. Hunt, Ashley W. Blom, Gulraj S. Matharu, Martyn L. Porter, Michael R. Whitehouse

**Affiliations:** 1 Musculoskeletal Research Unit, Bristol Medical School, University of Bristol & Southmead Hospital, Bristol, United Kingdom; 2 National Institute for Health Research Bristol Biomedical Research Centre, University Hospitals Bristol NHS Foundation Trust and University of Bristol, Bristol, United Kingdom; 3 Centre for Hip Surgery, Wrightington Hospital, Wrightington, Lancashire, United Kingdom; Monash University, AUSTRALIA

## Abstract

**Background and purpose:**

Over 1 million metal-on-metal hip replacements were implanted. Even well-functioning implants produce wear debris that can cause tissue damage, disseminate and cause DNA damage. We aimed to establish if there was an association between metal-on-metal hip replacement and the risk of subsequently developing cancer compared with alternative hip replacements.

**Methods:**

We performed a population based prospective longitudinal cohort study using data from the National Joint Registry linked to Hospital Episode Statistics (n = 403,881 patients). We examined the incidence of a new diagnosis of cancer in patients who received a metal-on-metal bearing in comparison with those who received a non metal-on-metal bearing. Kaplan-Meier estimates of time to first cancer diagnosis were used with Cox proportional hazards regression models to assess the effect on the time to cancer diagnosis for all cancer types, haematological, malignant melanoma, urinary tract cancers or prostate cancer in men.

**Results:**

The maximum follow up available was 11.8 years with 25% of patients followed up for more than 6.8 years (mean follow up 4.6 years; median 4.3; IQR 2.1–6.8; range 0.01–11.8). Analyses by gender that adjusted for age at primary and presence or absence of linked Welsh (PEDW) records showed no increase in the risk of developing cancer according to the bearing surface implanted for all cancers, haematological cancers, malignant melanoma, urinary tract cancers or prostate cancer in men. For patients receiving a second hip replacement, there was also no difference.

**Conclusion:**

We have demonstrated that there is currently no evidence of an increase in the risk of cancer following primary hip replacement according to the type of bearing material used. Although the risk of revision in metal-on-metal bearing hip replacements is higher, it is reassuring that the risk of a new diagnosis of cancer is not currently increased. Despite the long term follow up available in this study, the latency period for some cancers is very long and therefore continued monitoring is required to ensure no new patterns emerge that may indicate need for universal screening.

## Introduction

Total hip replacement (THR) is a commonly performed elective intervention for end stage hip osteoarthritis (OA) [1] that is both safe and cost effective [2]. Various material combinations can be used for the bearing couple in hip replacement, including metal-on-metal (MoM), metal-on-polyethylene (MoP), ceramic-on-polyethylene (CoP), ceramic-on-ceramic (CoC) and ceramic-on-metal (CoM) [1]. Wear and failure rates of THR are known to be higher in younger patients [1,3]. For this reason, MoM THR and hip resurfacing became popular between 2003 and 2008 due to the perception that preclinical tribological studies showing very low wear rates would translate to lower implant failure rates [4,5] and that the larger femoral head sizes that could be used would lead to lower risk of dislocation [6].

Over 1 million MoM hip replacements were implanted worldwide; by 2008 one-third of all hip replacements performed in the USA and 14% of those performed in England and Wales were MoM [7]. These devices experienced unexpectedly high failure rates [8,9], leading to large numbers of excess revisions [7] and most patients with an MoM bearing requiring regular follow-up. In June 2017 the Medicines and Healthcare Products Regulatory Agency (MHRA) updated its guidance and recommended more intensive surveillance for all MoM hip replacement patients [10]. This more intensive follow-up may reflect the MHRA’s continued and serious concerns about the long-term systemic effects of ions released from MoM hip replacements, which are largely unknown [11,12]. Other large cohort studies have shown no association between MoM hip replacements and the risk of cardiac disease in the medium term, another potential deleterious effect of metal ion release [13,14].

In vivo, all types of hip replacement release metal ions and particles; cobalt and chromium are two of the most common. Stemmed MoM and MoP hip replacements may release metal ions from the modular stem/head interface as well as the bearing surface [15–17]. These metals can deposit in the local tissues as particles, or be transported in the blood as ions. These metals can therefore be found in numerous organs including the kidney, bladder, ureter, liver, spleen, bone marrow, and lymph nodes [12,18,19]. Studies have shown that hip replacement patients have an increased risk of DNA damage to blood lymphocytes compared with controls [20]. Furthermore, in the occupational setting, an established relationship exists between high metal ion exposure and the development of certain cancers [12]. Although there is a theoretical increased risk of cancer in all patients receiving hip replacement, MoM hip replacements produce much higher concentrations of metal ions in the blood compared with conventional hip replacements, especially if the MoM hip replacement is not functioning optimally [21,22]. Hexavalent chromium (Cr^6+^) is a known carcinogen [23]. The concentrations of cobalt and chromium in the blood even in the case of well-functioning MoM hip replacements are capable of causing DNA damage across cellular barriers [24]. Therefore understandable public health concerns exist about whether the many patients worldwide with MoM hip replacement may have an increased risk of developing cancer secondary to metal ion exposure. Previous meta-analysis has identified an increased risk of prostate cancer in men and melanomas in patients that had undergone hip replacement compared to the general population [25]. This study also identified an increasing risk of urinary tract cancers over time after hip replacement, which may be due to the renal excretion of metal ions [26]. The exposure of haematopoietic cells to metal ions raises concerns over the risk of developinag haematological malignancies in the longer term [27], a phenomenon that has been observed in knee replacements [28] where there is also exposure to increased levels of cobalt and chromium [29].

Currently large cohort studies have suggested that patients with MoM hip replacement do not have an increased risk of cancer compared with hip replacements with other bearing surfaces [11,30,31]. However these studies have been limited to cohorts of up to 40,000 and also by short-term follow-up (mean follow-up 3.0–4.6 years). It is therefore important to establish if there is an increased risk of cancer in patients who have received a MoM hip replacement at longer term follow up. There is a known selection effect for patients undergoing elective interventions such as hip replacement and thus over time, have a lower mortality rate than an age and gender-matched population [32]. This may result in imbalances in the risk of developing or having already developed cancer between those that undergo hip replacement and those that do not, hence the best comparator when considering high-risk implants, is patients who underwent a hip replacement with alternative, lower-risk, implants [7,11].

Using data from the National Joint Registry of England, Wales, Northern Ireland and the Isle of Man (NJR) which is the largest joint registry worldwide, we investigated the association between the risk of being diagnosed with cancer following hip replacement for patients who received MoM bearings compared with those receiving non-MoM bearings.

## Methods

### Patients and data sources

**Fig 1:**
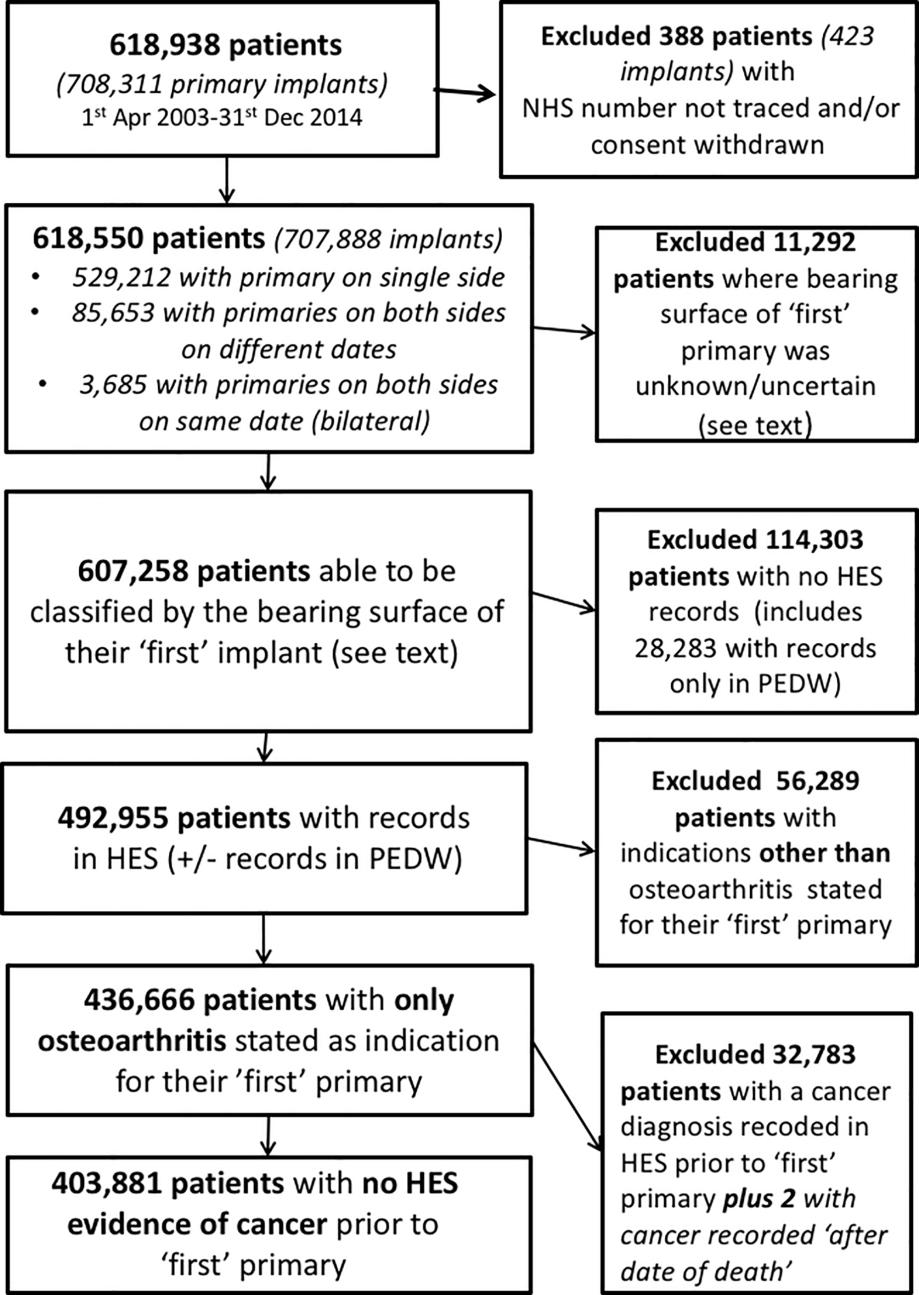
Patient population and exclusions from the study.

Data were obtained from the NJR, a comprehensive prospective national joint registry which collects information on primary and revision joint replacements. Data capture in the NJR is approximately 95% for primary hip replacements [1]. 708,311 primary hip replacements were reported for 618,938 patients between the 1st April 2003 and 31st December 2014, as described in Part 3 of the NJR 12th Annual Report [33]. Patients who underwent hip replacement in Northern Ireland had been excluded from the data set as there was no tracing service available for them. Patient inclusions and stepwise exclusions are detailed below and summarised in Fig 1.

Analyses were patient, rather than hip replacement, based. 388 patients were excluded because their NHS number was untraceable and/or consent was withdrawn. Of the remaining 618,550 patients 529,212 had a single primary operation, 3,685 patients had bilateral simultaneous operations (both hips replaced on the same day) and the remaining 85,653 had a second primary hip replacement performed at a later date. The patients were classified into groups according to the bearing surface materials of the first implant: stemmed MoM THR, MoM hip resurfacing, stemmed non-MoM THR (‘other’) and ‘uncertain’ bearing surface. Those patients (n = 3,685) who underwent bilateral procedures were classified as a ‘worst case’ scenario. If one or both implants were MoM, then they were assigned to the MoM group; if one or both implants were MoM resurfacings and, in the case of a resurfacing on one side, the other side was known not to be MoM, they were assigned to ‘resurfacing’ (S1 Table).

The 11,292 patients in whom the bearing surface was unknown were excluded from further analysis leaving a cohort of 607,258 patients for analysis. 84,050 patients in this cohort went on to receive a second primary hip replacement on the contralateral side in the period of interest. The second hip replacement was not consistently the same bearing surface as the first hip replacement (S2 Table); we aimed to explore the effect of any MoM bearing implanted at the second hip replacement in a later analysis.

The above data was linked to Hospital Episode Statistics (HES) inpatient records. Linkage was conducted by Northgate on behalf of the NJR. The HES extract consisted of all inpatient records for National Health Service funded care in England, from the 1998/99 financial year up to the end of 2014. From the HES inpatient records, we searched all ICD codes entered to find the first documented evidence of any cancer, excluding skin cancers (other than malignant melanoma) and we recorded the date of this first evidence. We similarly separately searched for the first documented diagnosis of each of the following: any haematological cancer (lymphoma, leukaemia, myeloma), melanoma (including melanoma in situ), urinary cancer (bladder, ureter or kidney) and prostate cancer (in men), which are suspected of being related to metal ion exposure [11], similarly defining the first date for each. A full list of the ICD codes that were used to define these is given in Table 1.

**Table 1:** ICD codes included in analysis.

	Included ICD 10 codes (any code beginning with)	Notes
**Any cancer**	C	Including C792 but excluding C44
	D00 to D09 inclusive	Excluding D04
	D37 to D48 inclusive	
**Any haematological cancer (lymphoma, leukaemia, myeloma)**	C81 to C86 inclusive	
	C88 to C96 inclusive	
	D45 to D47 inclusive	
	C77	Unspecified malignant neoplasm of lymph nodes
**Any malignant melanoma**	C43	
	D03	Melanoma in situ
**Any prostate cancer**	C61	In men
	D075	
	D400	
**Any urinary cancer (bladder, ureter, kidney)**	C64 to C68 inclusive	
	C790 to C791 inclusive	
	D090 to D091 inclusive	
	D41	

Not all of the NJR patients had episodes in HES; as previously described [34], they may not have had HES records if they had had only independently-funded procedures, or only had in-patient admissions in Wales. We sought to supplement the data using data from the Patient Episode Database for Wales (PEDW) over the same period, but many of the dates on our extract were incomplete making it impossible to define dates of first cancers. We did however note whether the patient did have PEDW records, in addition to or instead of HES.

Of the 607,258 patients above, we excluded 114,303 patients with no HES records at all, leaving 492,955 patients for analysis. Of these, 5,484 of these also had records in PEDW. Given this is a potential source of bias (since cancer diagnoses thereby may be missed, if in PEDW and not in HES, or appear to be later if in both but reported earlier in PEDW than in HES), this information (whether there were episodes in PEDW or not) was retained as an instrumental variable.

Amongst the remainder, 28,283 (5%) had records only in PEDW and these, together with 86,020 with records in neither HES nor PEDW were excluded.

We believed that some patients’ may have had cancer reported for the first time at death. The Office for National Statistics (ONS) cause(s) of death in those who died were available for those who could be HES-linked; we searched all listed causes of death to find any of the relevant cancers defined above, so enhancing the information already ascertained.

Analysis was restricted to cases where the first hip replacement was performed for a diagnosis of OA only, leaving 436,666 patients for final analysis. Due to the popularity of MoM bearings in the earlier years of the NJR which subsequently declined [1] following the identification of higher failure rates in MoM bearings [8,9], we noted the median year of implantation varied by bearing (stemmed MoM THR: 2008; resurfacing: 2007; other (non-MoM) THR: 2010) leading us to consider year of primary as a potential covariate.

As a final step we excluded 32,783 patients who were found to have had a first diagnosis of cancer before the date of their primary hip replacement (or up to 7 days afterwards), together with 2 cases with a date of death that preceded the date of first diagnosis of cancer. We noted the proportions excluded at this step reflected the different age/gender demographics of the three comparison subgroups (S3 Table)

Our final analysis, therefore, was based on 403,881 patients.

### Statistical methods

We used Kaplan-Meier estimates of first cancer diagnosis, together with a series of Cox ‘proportional hazards’ regression models to assess the effects of the bearing surface material of the first primary hip replacement on the time to a cancer diagnosis, adjusting for age at primary (grouped 45–49, 50–54, 55–59, 60–64, 65–69, 70–74, 75–79, 80–84 and 85+ years), American Society of Anesthesiologists Physical Classification System grade (ASA; grouped 1, 2, 3, 4/5, see below) and whether or not we could find records in PEDW, followed by graphical checks of proportional hazards. Males and females were analysed separately due to the different cumulative risk of developing a cancer between genders. This was repeated for each of the specified cancers defined above.

For all-cause cancer, we also (i) explored the competing risk of death from other causes (other than the defined cancers) using Fine and Gray analysis [35] and (ii) conducted a series of sensitivity analyses in which, in separate analyses for stemmed MoM case and MoM hip resurfacings, for men and women, we randomly selected 1:1 and 1:4 matched samples of patients from amongst the (larger) group of stemmed non-MoM cases. Increasing the ratio of matches in a cohort study design increases precision, but there is a risk of introducing bias, we therefore report the results of both for transparency [36]. Fine and Gray analysis was used in this context as it is possible that the exposure of interest was related to the risk of mortality (i.e. the competing risk of death may be informative or at least partially informative depending upon the diagnostic accuracy of the cause of death which can not be established in this cohort). Matching was done on the basis of grouped age, ASA, whether or not the patient had records in PEDW, the year of primary surgery (+/- one year) and the fifth of area deprivation. The latter were based on the Indices of Multiple Deprivation (IMDs) associated with the patient’s ‘small area’ of residence, as documented in their HES records, as close as possible to the date of the primary operation. We used the fifths of the ranked areas, thus 1 meant that the patient was living in one of the 20% most deprived areas, 5 the 20% least deprived. The matchings ensured perfectly matched comparisons, albeit with smaller sample sizes; the random sampling, furthermore, helped to balance groups in respect of unmeasured variables within the matched sets. There were fewer cases for 1:4 matching than 1:1 as sampling was without replacement. Cox ‘proportional hazards’ regression models were used to compare the stemmed MoM or the MoM hip resurfacing groups with their matched stemmed non-MoM counterparts, using a robust clustered variance to allow for the matched sets.

Finally, there were cases that went on to receive a hip replacement on the contralateral side. In these cases, we observed that the second implant received was not conditional on the basis of the first implant received and as such, there were a variety of possible exposure patterns and times at risk amongst the cohort. As the exposure of interest was whether a patient had received a stemmed MoM THR or MoM hip resurfacing, we therefore explored how the risk of all-cause cancer may have changed amongst the ‘other’ group if/when a patient had a second implant that was either a stemmed MoM THR or MoM hip resurfacing. These latter effects were modelled using time-dependent covariates.

For type-specific cancer, we looked in turn at each of four specific types of cancer: (a) haematological cancer, (b) malignant melanoma, (c) urinary tract cancer and (d) prostate cancer (in men). Cox proportional hazard models were used to assess the time to a new diagnosis of a specific cancer. For this analysis, follow up was censored to be either the end of 2014 or the (earlier) date of death if the patient had died from either non-cancer causes or from any cancer of a different type. In those who developed the cancer of interest, the date of the first evidence of this type of cancer was taken whether that was first documented when the patient was alive or was first documented as a cause of death. For those in whom the cancer was first diagnosed as a cause of death, if there had been one of the other cancer types diagnosed prior to the cancer of interest being documented, the case was censored at this earlier time point, because the risk of the patient developing the cancer of interest may have been altered by their having had a cancer of a different type.

The statistical analysis used the software package Stata version 14.2 (StataCorp LLC, Texas, 1985–2015).

## Results

### Effect of the bearing type in the first primary hip replacement on the risk of a subsequent cancer diagnosis

The demographics of our groups for comparison are shown in Table 2.

**Table 2:** Characteristics of final groups of primary hip replacement (n = 403,881) for analysis by bearing type.

Bearing type for first primary hip	Number	Age at first primary (years) Median (IQR)	Percentage of males	ASA (at first primary)			
				1 (normal healthy patient)	2 (mild systemic disease)	3 (severe systemic disease)	4–5 (severe systemic disease that is threat to life, or greater)
**MoM**	17,351	64 (57–71)	50.9%	26.1%	62.7%	10.8%	0.4%
**Resurfacing**	18,565	55 (49–60)	70.6%	46.3%	50.4%	3.2%	0.1%
**Other**	367,965	70 (63–77)	38.4%	15.0%	70.1%	14.4%	0.5%

In univariable analysis (Kaplan-Meier), the cumulative probability of being diagnosed with a cancer was higher in males than females (S1 Fig), older age groups (S2 and S3 Figs) and in higher ASA grades (S4 Fig). Further analyses were therefore adjusted for these variables. The cumulative risk of diagnosis of cancer did not differ overall by the year of primary (S5 Fig).

**Fig 2:**
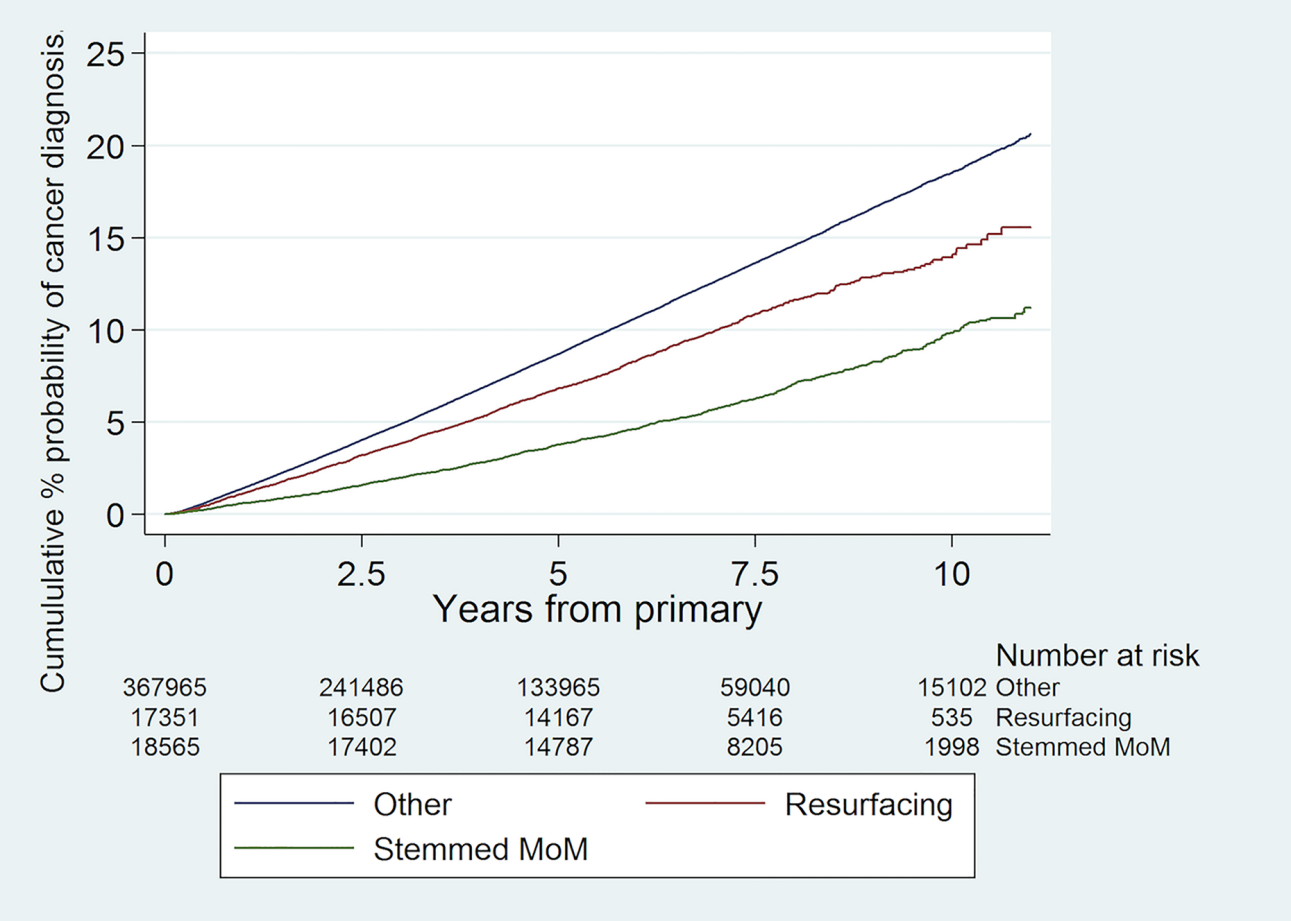
Cumulative risk of a new diagnosis of any type of cancer following primary hip replacement by bearing type with numbers at risk shown.

The cumulative percentage probability of being diagnosed with a cancer for the first time after primary hip replacement by bearing type and the numbers at risk are shown in Fig 2. ‘Unadjusted’ (i.e. without adjustment for the confounding variables above) Cox proportional hazards regression models demonstrated a lower risk of a subsequent diagnosis of cancer following primary hip replacement in patients who received a stemmed MoM THR or hip resurfacing in comparison to stemmed non-MoM THR in both men and women (Table 3); these effects attenuated with adjustment for age and ASA grade. Further adjustment for whether a patient had a record in PEDW or not did not affect the findings. The results were very similar when we included year of primary. In this model, the year of primary was not related to outcome, but this was difficult to assess given its relationship with length of follow up; we revisited year of primary in our sensitivity analysis below.

**Table 3:** Hazard Rate Ratios (HRR) for being diagnosed with any type of cancer according to bearing type following primary hip replacement in men (n = 163,111; 14,825 were subsequently diagnosed with cancer) and women (n = 240,765; 17,040 were subsequently diagnosed with cancer).

Bearing type for first primary hip	(i) Unadjusted		(ii) Adjusted for age and ASA		(iii) Adjusted for age, ASA and whether patient had a record in PEDW	
	HRR [95% CI]	p-value	HRR [95% CI]	p-value	HRR [95% CI]	p-value
**Men**						
**Other**	1 [referent]		1 [referent]		1 [referent]	
**MoM**	0.71 [0.67–0.76]	p<0.001	0.99 [0.92–1.06]	p = 0.737	0.99 [0.92–1.05]	p = 0.687
**Resurfacing**	0.38 [0.35–0.40]	p<0.001	0.90 [0.83–0.97]	p = 0.008	0.90 [0.83–0.97]	p = 0.007
**Women**						
**Other**	1 [referent]		1 [referent]		1 [referent]	
**MoM**	0.76 [0.71–0.82]	p<0.001	0.93 [0.86–1.00]	p = 0.050	0.93 [0.86–1.00]	p = 0.043
**Resurfacing**	0.51 [0.46–0.57]	p<0.001	0.99 [0.88–1.10]	p = 0.803	0.99 [0.88–1.10]	p = 0.815

Table 4 shows Fine and Gray sub-hazard rate ratios which account for the competing risk of death from other causes (than deaths from the cancers of interest).

**Table 4:** Sub-hazard Rate Ratios (SHR) for being diagnosed with any type of cancer according to bearing type following primary hip replacement in men (n = 163,111; 14,825 were subsequently diagnosed with cancer; 9,524 ‘competing’ deaths) and women (n = 240,765; 17,040 were subsequently diagnosed with cancer; 13,999 ‘competing’ deaths).

Bearing type for first primary hip	(i) Unadjusted		(ii) Adjusted for age, ASA and whether patient had a record in PEDW	
	SHR [95% CI]	p-value	SHR [95% CI]	p-value
**Men**				
**Other**	1 [referent]		1 [referent]	
**MoM**	0.73 [0.69–0.78]	p<0.001	0.99 [0.93–1.06]	p = 0.743
**Resurfacing**	0.40 [0.37–0.43]	p<0.001	0.92 [0.85–0.99]	p = 0.027
**Women**				
**Other**	1 [referent]		1 [referent]	
**MoM**	0.78 [0.73–0.84]	p<0.001	0.93 [0.86–1.00]	p = 0.055
**Resurfacing**	0.54 [0.49–0.60]	p<0.001	1.00 [0.90–1.21]	p = 0.931

Table 5 summarises our sensitivity analyses, for the stemmed MoM cases and MoM hip resurfacing primaries, respectively, comparing each with stemmed non-MoM implants, using matched samples on the basis of age group, ASA, whether or not the patient had records in PEDW, year of primary (+/- one year) and IMD fifths. IMD fifths were available for 98.9% of the whole cohort.

**Table 5:** Hazard Rate Ratios (HRR) with: (i) 1:1 matching for being diagnosed with any type of cancer following stemmed MoM in men (a: n = 17,450; 1,879 were subsequently diagnosed with cancer) and women (c: n = 16,930; 1,437 were subsequently diagnosed with cancer), and MoM hip resurfacings in men (b: n = 23,722; 1,529 were subsequently diagnosed with cancer) and women (d: n = 10,672; 704 were subsequently diagnosed with cancer). (ii) 1:4 matching for being diagnosed with any type of cancer following stemmed MoM in men (a: n = 41,000; 4,462 were subsequently diagnosed with cancer) and women (c: n = 41,815; 3,704 were subsequently diagnosed with cancer), and MoM hip resurfacings in men (b: n = 38,350; 2,684 were subsequently diagnosed with cancer) and women (d: n = 20,425; 1,375 were subsequently diagnosed with cancer).

Bearing type for first primary hip	(i) 1:1 matching			(ii) 1:4 matching		
	Total number of cases (number who developed cancer)	HRR [95% CI]	p-value	Total number of cases (number who developed cancer)	HRR [95% CI]	p-value
**Men**						
**(a)**	17,450 (1,879)			41,000 (4,462)		
**Other**		1 [referent]			1 [referent]	
**MoM**		0.98 [0.89–1.07]	p = 0.592		1.00 [0.93–1.07]	p = 0.966
**(b)**	23,722 (1,529)			38,350 (2,684)		
**Other**		1 [referent]			1 [referent]	
**Resurfacing**		0.88 [0.80–0.97]	p = 0.013		0.84 [0.76–0.92]	p<0.001
**Women**						
**(c)**	16,930 (1,437)			41,815 (3,704)		
**Other**		1 [referent]			1 [referent]	
**MoM**		0.98 [0.88–1.09]	p = 0.702		0.92 [0.85–1.00]]	p = 0.060
**(d)**	10,672 (704)			20,425 (1,375)		
**Other**		1 [referent]			1 [referent]	
**Resurfacing**		0.95 [0.83–1.10]	p = 0.521		0.97 [0.85–1.11]	p = 0.697

For the men, 8,725 of the 8,749 stemmed MoM (99.7%) could be matched to one ‘other’ type of implant (see Table 5(A) column (i)) and 8,200 (93.7%) could be matched to exactly 4 (see (a) (ii)). In the second analysis, 11,861 of the 12,927 resurfacings (91.8%) could be matched to one ‘other’ type of implant (see (b) column (i)). The 8.2% of resurfacings that could not be matched tended to be younger, with better ASA, more likely to have records in PEDW, earlier primaries and less deprived than the 91.8% that were matched, however their risks of cancer did not differ statistically significantly from the unmatched group when these factors were taken into account. Only 7,970 (59.3%) of the 12,927 resurfacings could be matched to exactly 4 ‘other’ implants (see (b) (ii)).

For the women, 8,465 of the 8,472 stemmed MoM (99.9%) could be matched to one ‘other’ type of implant (see Table 5(C) (i)) and 8,363 (98.7%) could be matched to exactly 4 implants ((c) (ii)). 5,336 (99.0%) of the 5,390 resurfacings could be matched to one ‘other’ type of implant (see (d) (i)). Only 4,085 (78.4%), however, could be matched to exactly 4 ((d) (ii)).

The results in Table 5 accord with those in column (iii) of Table 3 but using matched samples. We were not able to find any evidence from these analyses that the risk of cancer was increased in stemmed MoM or MoM hip resurfacings.

### Effect of the bearing type in the second primary hip replacement on the time to a subsequent cancer diagnosis

Amongst the 367,965 who initially received a stemmed non-MoM THR (‘other’) for OA only, 52,111 went on to have a second implant. The majority of which (50,940) were documented to have received similar (i.e. ‘other’) bearing types. However, 474 received a stemmed MoM THR and 40 a resurfacing, whilst in a further 657 cases the bearing type was uncertain.

We modelled the time to cancer diagnosis, again from the initial primary implant, to look at effects of later primaries that were (i) ‘other’ types, (ii) stemmed MoM, (iii) Resurfacing and (iv) uncertain types using those in who a second primary had not been performed as the control. These were time-dependent effects in that they became operative only after a second primary implant had been performed. Analysis was performed for men and women separately and adjustment made for age, ASA and whether patients also had a record in PEDW (Table 6).

**Table 6:** Cox proportional hazards regression models of the relative effects on time to cancer diagnosis of the bearing types of a second (contralateral) primary hip replacement, for patients who received stemmed non-MoM THR (other) at first primary in men (n = 141,179; 13,076 were subsequently diagnosed with cancer) and women (n = 259,858; 15,958 were subsequently diagnosed with cancer).

	(i) Unadjusted		(ii) Adjusted for age, ASA and whether patient had a record in PEDW	
	HRR [95% CI]	p-value	HRR [95% CI]	p-value
**Men**				
**No second primary performed**	1			
**Effect associated with a second primary that has bearing type:**				
**Other**	0.89 [0.84–0.94]	p<0.001	0.97 [0.92–1.03]	p = 0.377
**MoM**	1.01 [0.70–1.47]	p = 0.948	1.24 [0.86–1.80]	p = 0.253
**Resurfacing**	0.46 [0.11–1.82]	p = 0.267	0.82 [0.21–3.28]	p = 0.780
**Uncertain**	0.61 [0.37–1.00]	p = 0.050	0.68 [0.42–1.11]	p = 0.126
	Comparison between the hazard rates associated with the type of second primary in model (ii):		‘Other’ vs. ‘MoM’ p = 0.204 ‘Other’ vs. ‘Resurfacing’ p = 0.809 ‘MoM’ vs. ‘Resurfacing’ p = 0.572	
**Women**				
**No second primary performed**	1 [referent]		1 [referent]	
**Effect associated with a second primary that has bearing type:**				
**Other or resurfacing**	0.89 [0.85–0.94]	p<0.001	0.95 [0.91–1.00]	p = 0.060
**MoM**	0.77 [0.52–1.15]	p = 0.203	0.89 [0.60–1.33]	p = 0.571
**Uncertain**	1.14 [0.83–1.57]	p = 0.408	1.24 [0.90–1.71]	p = 0.181
	Comparison between the hazard rates associated with the type of second primary in model (ii):		‘Other’ or ‘Resurfacing’ vs. ‘MoM’ p = 0.744	

In men, we found no evidence that subsequent stemmed MoM THR or resurfacings were associated with higher cancer incidence, although the numbers were small, particularly for the latter. Given there may also have been patient selection as to whether or not the patient got a second implant at all, we compared the respective coefficients (not shown) for the effects of stemmed MoM THR and resurfacing in the adjusted model with those for a subsequent ‘other’ bearing type, we found no statistically significant differences. In women there were no cancers found after the resurfacing procedure and we made a pragmatic decision to combine this group with the ‘other’ group; there was no evidence that the stemmed MoM THR group had a higher risk of cancer.

### Effect of the bearing type in the first primary hip replacement on the risk of a subsequent diagnosis of specific types of cancer

These analyses were based on the 403,881 patients with no cancer documented prior to their first primary in NJR. 348,491 of these patients were alive at the end of follow up with no cancer diagnosis having been made and 23,524 died of causes other than cancer (with no preceding cancer diagnosis).

#### (a) Haematological cancer

When the haematological cancers were considered, 5,062 patients were diagnosed with a haematological cancer where this was the first cancer detected following the primary hip replacement. There were 26,804 cases in whom a different type of cancer was the first cancer detected following the primary hip replacement.

Unadjusted analysis suggested a lower risk of haematological cancers in patients who received a stemmed MoM THR or hip resurfacing but this effect attenuated with adjustment in men and women (Table 7).

**Table 7:** Cox proportional hazards regression models of the risk of being diagnosed with a haematological cancer following primary hip replacement by bearing type for men (n = 163,111; 2,109 were subsequently diagnosed with a haematological cancer) and women (n = 240,765; 2,953 were subsequently diagnosed with a haematological cancer).

Bearing type for first primary hip	(i) Unadjusted		(ii) Adjusted for age and ASA		(iii) Adjusted for age, ASA and whether patient had a record in PEDW	
	HRR [95% CI]	p-value	HRR [95% CI]	p-value	HRR [95% CI]	p-value
**Men**						
**Other**	1 [referent]		1 [referent]		1 [referent]	
**MoM**	0.64 [0.53–0.77]	p<0.001	0.89 [0.74–1.07]	p = 0.211	0.89 [0.74–1.07]	p = 0.737
**Resurfacing**	0.43 [0.36–0.51]	p<0.001	0.96 [0.79–1.17]	p = 0.706	0.96 [0.79–1.17]	p = 0.702
**Women**						
**Other**	1 [referent]		1 [referent]		1 [referent]	
**MoM**	0.79 [0.67–0.95]	p = 0.010	1.00 [0.84–1.19]	p = 0.995	1.00 [0.84–1.19]	p = 0.977
**Resurfacing**	0.35 [0.26–0.47]	p<0.001	0.73 [0.53–1.00]	p = 0.050	0.73 [0.53–1.00]	p = 0.051

#### (b) Malignant melanoma

When malignant melanoma was considered, 1,172 patients were diagnosed with a malignant melanoma where this was the first cancer detected following the primary hip replacement. There were 30,694 cases in whom a different type of cancer was the first cancer detected following the primary hip replacement.

Unadjusted analysis suggested a lower risk of malignant melanoma in patients who received a hip resurfacing in men but this effect attenuated with adjustment (Table 8). There were no statistically significant differences according to bearing type seen in stemmed MoM THR in men nor in stemmed MoM THR or resurfacing in women (Table 8).

**Table 8:** Cox proportional hazards regression models of the risk of being diagnosed with malignant melanoma following primary hip replacement by bearing type for men (n = 163,111; 576 were subsequently diagnosed with malignant melanoma) and women (n = 240,765; 596 were subsequently diagnosed with malignant melanoma).

Bearing type for first primary hip	(i) Unadjusted		(ii) Adjusted for age and ASA		(iii) Adjusted for age, ASA and whether patient had a record in PEDW	
	HRR [95% CI]	p-value	HRR [95% CI]	p-value	HRR [95% CI]	p-value
**Men**						
**Other**	1 [referent]		1 [referent]		1 [referent]	
**MoM**	0.87 [0.64–1.18]	p = 0.359	1.13 [0.83–1.54]	p = 0.441	1.13 [0.83–1.54]	p = 0.451
**Resurfacing**	0.38 [0.26–0.54]	p<0.001	0.75 [0.51–1.12]	p = 0.158	0.75 [0.51–1.11]	p = 0.156
**Women**						
**Other**	1 [referent]		1 [referent]		1 [referent]	
**MoM**	0.81 [0.55–1.19]	p = 0.287	0.92 [0.62–1.36]	p = 0.669	0.92 [0.62–1.36]	p = 0.672
**Resurfacing**	0.65 [0.39–1.06]	p = 0.085	0.95 [0.56–1.62]	p = 0.855	0.95 [0.56–1.62]	p = 0.854

#### (c) Urinary tract cancer

When urinary tract cancers were considered, 2,927 patients were diagnosed with a urinary tract cancer where this was the first cancer detected following the primary hip replacement. There were 28,939 cases in whom a different type of cancer was the first cancer detected following the primary hip replacement.

Unadjusted analysis suggested a lower risk of urinary tract cancer in patients who received a stemmed MoM THR or hip resurfacing in men or a hip resurfacing in women but this effect attenuated with adjustment (Table 9). There were no statistically significant differences in the unadjusted or adjusted analyses of stemmed MoM THR in women (Table 9).

**Table 9:** Cox proportional hazards regression models of the risk of being diagnosed with a urinary tract cancer following primary hip replacement by bearing type for men (n = 163,111; 1,849 were subsequently diagnosed with a urinary tract cancer) and women (n = 240,765; 1,078 were subsequently diagnosed with a urinary tract cancer).

Bearing type for first primary hip	(i) Unadjusted		(ii) Adjusted for age and ASA		(iii) Adjusted for age, ASA and whether patient had a record in PEDW	
	HRR [95% CI]	p-value	HRR [95% CI]	p-value	HRR [95% CI]	p-value
**Men**						
**Other**	1 [referent]		1 [referent]		1 [referent]	
**MoM**	0.73 [0.60–0.88]	p = 0.001	1.06 [0.88–1.28]	p = 0.510	1.06 [0.88–1.28]	p = 0.535
**Resurfacing**	0.35 [0.28–0.43]	p<0.001	0.97 [0.77–1.23]	p = 0.814	0.97 [0.77–1.22]	p = 0.801
**Women**						
**Other**	1 [referent]		1 [referent]		1 [referent]	
**MoM**	0.79 [0.59–1.05]	p = 0.108	1.04 [0.78–1.39]	p = 0.785	1.03 [0.77–1.38]	p = 0.819
**Resurfacing**	0.30 [0.18–0.51]	p<0.001	0.78 [0.45–1.35]	p = 0.370	0.78 [0.45–1.35]	p = 0.375

#### (d) Prostate cancer in men

When cancer of the prostate in men was considered, 138,762 men remained alive at the end of follow up with no diagnosis of a cancer. 9,524 men had died of a cause other than cancer before the end of follow up (with no prior cancer diagnosis). 4,061 men were diagnosed with prostate cancer where this was the first cancer detected following the primary hip replacement. There were 10,764 cases in whom a different type of cancer was the first cancer detected following the primary hip replacement.

Unadjusted analysis suggested a lower risk of prostate cancer in men who received a stemmed MoM THR or hip resurfacing in men (Table 10). In stemmed MoM THR, this effect attenuated with adjustment for age, ASA and whether the patient had a record in PEDW or not. In the case of resurfacing, this difference remained statistically significant despite this adjustment, with a higher risk in the resurfacing group. The risk of prostate cancer differed markedly between the age groups and in this instance their hazard rates were not proportional; further analysis that stratified by age (i.e. assumed differently shaped baseline hazards for the age groups) attenuated the resurfacing group effect (column (iv) Table 10).

**Table 10:** Cox proportional hazards regression models of the risk of being diagnosed with prostate cancer following primary hip replacement by bearing type for men (n = 163,111; 4,061 were subsequently diagnosed with prostate cancer).

Bearing type for first primary hip	(i) Unadjusted		(ii) Adjusted for age and ASA		(iii) Adjusted for age, ASA and whether patient had a record in PEDW		(iv) Adjust for ASA and whether patient records in PEDW (instrumental variable) and stratified by age	
	HRR [95% CI]	p-value	HRR [95% CI]	p-value	HRR [95% CI]	p-value	HRR [95% CI]	p-value
**Other**	1 [referent]		1 [referent]		1 [referent]		1 [referent]	
**MoM**	0.65 [0.57–0.74]	p<0.001	0.91 [0.80–1.04]	p = 0.179	0.91 [0.80–1.04]	p = 0.164	0.90 [0.79–1.03]	p = 0.126
**Resurfacing**	0.45 [0.39–0.51]	p<0.001	1.16 [1.01–1.33]	p = 0.037	1.16 [1.01–1.33]	P = 0.040	1.11 [0.97–1.28]	p = 0.141

## Discussion

In a large national prospective registry analysis of 403,881 primary hip replacements who received either a stemmed MoM THR, MoM resurfacing or stemmed non-MoM THR, 31,866 patients had a new diagnosis of cancer after their primary hip replacement was performed. The cumulative risk of cancer was higher in males, older patients, and those with greater ASA grades. When analyses were adjusted for these variables, the risk of diagnosis of any type of cancer was not statistically significantly higher for MoM bearings when compared to non-MoM bearings. For those that went on to receive a second hip replacement, there was also no difference in the risk of developing a cancer. When specific types of cancer were considered in men and women (haematological cancers, malignant melanoma and urinary tract cancers), there was no statistically significant effect on the risk of developing a cancer according to the type of bearing. When prostate cancers in men were considered, there initially appeared to be a higher risk in patients who had received an MoM resurfacing compared to stemmed non-MoM THR, however given the non-proportionality of the hazard of developing prostate cancer by age group an age stratified analysis was performed which demonstrated that this effect was not statistically significant.

Large cohort studies have largely concluded that MoM hip replacement patients do not have an increased risk of cancer in the short-term compared with conventional hip replacement patients [11,30,31,37,38]. The initial analysis of over 40,000 MoM hip replacement patients from the NJR for England and Wales observed that the risk of all cancer diagnoses, and specifically malignant melanoma, prostate, renal tract, and haematological cancers, were similar to that in conventional hip replacement patients (mean 3.0 year follow-up) [11]. A study of over 10,000 MoM hip replacement patients from the Finnish Arthroplasty Register reported no differences in the risk of cancer compared with both the normal population and conventional hip replacement patients (mean 3.6 year follow-up) [31] and this was maintained in the medium term (mean 7.4 year follow-up) [39]. Another study from England using linked data on over 11,000 hip replacement patients from the NJR and General Practice databases also demonstrated MoM hip replacement patients did not have an increased risk of cancer compared with conventional hip replacement patients (mean 3.2 year follow-up) [37]. Similar observations were reported for patients in Scotland, however this only included 1,317 MoM hip replacement patients with short-term follow-up [38]. Our findings extend the follow up period available in previous analyses and demonstrate that there is no increased risk of developing any cancer, haematological cancer, malignant melanoma, cancer of the urinary tract, or prostate cancer in men following primary hip replacement in association with the bearing surface material implanted.

Study strengths include using linked data from the worlds largest arthroplasty registry. The specific cohort studied was a large, comprehensive and representative sample of patients undergoing primary hip replacement in the NHS over 12 years. The patients receiving hip replacements, indications for surgery and implants used are broadly similar between the NJR and other large national registries meaning that our results should be generalisable to other populations, however the incidence of cancers does vary geographically meaning there may be a baseline difference in risk between populations. Patients receiving different types of bearing surface may have been subject to a selection effect and were not randomly allocated meaning that we can perform detailed analyses of association with the risk of developing cancer following an index procedure but can directly establish if the bearing type selected was a causative factor. Some cancer types have very long latency periods meaning that longer term surveillance extending into decades will be required in order to establish if there is a late effect on the risk of developing a cancer. The pattern of usage of the different bearing types has not been consistent across the period studied. MoM bearings underwent huge growth in their use on the early period of the NJR but once their unacceptably high failure rates in comparison to contemporary alternatives had been established, their use declined rapidly. Patients receiving the different bearing types studied have therefore been at risk for variable periods with different periods of coverage in HES prior to implantation. We examined the risk according to the year of primary hip replacement and found that there was no difference according to the year group and therefore we did not further adjust for this. The modular junctions of hip replacements are another potential source of metal ion release [16,17,40] with complications secondary to this even observed in implants without bearing surfaces [41] and in some cases, it is felt that this may contribute more than wear at the bearing surface [15]. Given the variation of release according to design, implants factors, demographics and materials [16], we have not been able to fully explore this in our analyses but use non-MoM bearings as a control group for MoM bearings.

We have used a consistent method of identifying cancers using HES records. It is likely that we may have missed some cancer diagnoses as not every diagnosis of cancer will trigger an inpatient admission and hence be captured by HES. The use of the cause of death recorded by the ONS allows us to capture a further proportion of cancer diagnoses but even this may miss some cancer diagnoses when they were not associated directly with the cause of death or missed as secondary diagnoses. We are limited to capturing cancer diagnoses at the first time they are documented rather than when the cancer actually developed but this is a limitation common to all such studies of diagnoses following intervention. There is no evidence to suggest that these patterns would have differed between the different types of hip bearing types and hence it is reasonable to assume that any missing data is missing at random.

The risk of developing some cancers, such as prostate cancer in men, has large variation according to age and the risk is not constant. We therefore checked our models to establish where such variation existed and stratified by age in the analysis of prostate cancer which demonstrated that what at first appeared to be a statistically significant association attenuated. It is likely that patients who had developed a cancer prior to receiving a hip replacement would have had a different susceptibility to developing a subsequent cancer than those who had not and it is also likely that this susceptibility would have varied by cancer type. We therefore restricted our cohort to censor those that had a prior record of cancer preceding the primary hip replacement. This reduced the size of the cohort available for analysis but could have only have been avoided if we had comprehensive data available for all patients form birth through to the end point of the study, which was not available. It is possible that some patients had developed a cancer prior to receiving a primary hip replacement but this had either not been diagnosed or had not been captured in the datasets we examined. We have attempted to reduce this risk by using the presence of a record in PEDW or not as an instrumented variable but we can not fully control for this risk.

### Conclusions

It is reassuring that we have demonstrated no increased risk of cancer in association with the bearing surface material used in primary hip replacement. Although the failure rate of MoM bearings is higher, and therefore more patients are exposed to revision in this cohort, the risk of MoM patients developing a new diagnosis of cancer was not raised. This is reassuring for patients and clinicians and the follow up data presented here now extends into long term follow up. Despite this, the latency period of some cancers exceeds the follow up available even here and continued monitoring of this risk is required. These patients have not specifically been screened for cancers and if an increased risk of a particular type of cancer is demonstrated in the future, appropriate screening may be required to allow early detection. Future analyses would benefit from linkage to cancer registries to enrich the dataset and allow more in depth analysis, something that has not been possible to do at this stage.

## Supporting information

S1 Table Classification of type of first primary hip replacement by bearing surface. (DOCX)

S2 Table Bearing surface classification for first primary hip replacement and classification of second hip replacement bearing for 84,050 patients who had left and right primary hip replacements at different times. (DOCX)

S3 Table Year of primary hip replacement by bearing type. (DOCX)

S4 Table Proportion of patients with a record of a previous cancer diagnosis recorded in HES prior to primary hip procedure by bearing type. (DOCX)

S1 Fig Cumulative risk of a new diagnosis of any type of cancer following primary hip replacement by gender. (DOCX)

S2 Fig Cumulative risk of a new diagnosis of any type of cancer following primary hip replacement by age at first primary for males. (DOCX)

S3 Fig Cumulative risk of a new diagnosis of any type of cancer following primary hip replacement by age at first primary for females. (DOCX)

S4 Fig Cumulative risk of a new diagnosis of any type of cancer following primary hip replacement by ASA at the first primary hip replacement. (DOCX)

S5 Fig Cumulative risk of a new diagnosis of any type of cancer following primary hip replacement by year of primary. (DOCX)
