# Supplementary material for: The risk of developing cancer following metal-on-metal hip replacement compared with non metal-on-metal hip bearings: Findings from a prospective national registry “The National Joint Registry of England, Wales, Northern Ireland and the Isle of Man”
Source: PLoS One. 2018 Sep 20;13(9):e0204356. doi: 10.1371/journal.pone.0204356 (PMC6147563; doi:10.1371/journal.pone.0204356)
Supplement: S1 Table — (DOCX) [file pone.0204356.s001.docx]

### S1 Table. Classification of type of first primary hip replacement by bearing surface.

| **Type of hip replacement** | **Unilateral** | **Bilateral** | **Total** |
| --- | --- | --- | --- |
| **MoM**  **(MoM THR)** | 27,948 | 359 | 28,307 |

| **Resurfacing**  **(MoM hip resurfacing)** | 32,449 | 421 | 32,870 |
| --- | --- | --- | --- |

| **Other**  **(non-MOM THR)** | 543,273 | 2,808 | 546,081 |
| --- | --- | --- | --- |
| **Uncertain** | 11,195 | 97 | 11,292 |
| **Total** | 614,865 | 3,685 | 618,550 |
