# Supplementary material for: The risk of developing cancer following metal-on-metal hip replacement compared with non metal-on-metal hip bearings: Findings from a prospective national registry “The National Joint Registry of England, Wales, Northern Ireland and the Isle of Man”
Source: PLoS One. 2018 Sep 20;13(9):e0204356. doi: 10.1371/journal.pone.0204356 (PMC6147563; doi:10.1371/journal.pone.0204356)
Supplement: S2 Table — (DOCX) [file pone.0204356.s002.docx]

### S2 Table. Bearing surface classification for first primary hip replacement and classification of second hip replacement bearing for 84,050 patients who had left and right primary hip replacements at different times.

| **First hip replacement bearing** | **Second hip replacement bearing** | | | |  |
| --- | --- | --- | --- | --- | --- |
|  | **Other** | **MoM** | **Resurfacing** | **Uncertain** | **Total** |
| **MoM** | 3,028 | 2,384 | 84 | 54 | 5,550 |
| **Resurfacing** | 2,576 | 287 | 4,065 | 49 | 6,977 |
| **Other** | 69,910 | 656 | 71 | 886 | 71,523 |
| **Total** | 75,514 | 3,327 | 4,220 | 989 | 84,050 |
