# Supplementary material for: The risk of developing cancer following metal-on-metal hip replacement compared with non metal-on-metal hip bearings: Findings from a prospective national registry “The National Joint Registry of England, Wales, Northern Ireland and the Isle of Man”
Source: PLoS One. 2018 Sep 20;13(9):e0204356. doi: 10.1371/journal.pone.0204356 (PMC6147563; doi:10.1371/journal.pone.0204356)
Supplement: S3 Table — (DOCX) [file pone.0204356.s003.docx]

### S3 Table. Year of primary hip replacement by bearing type

| **Bearing type for first primary hip** | **Number** | **Year of primary** | | | |
| --- | --- | --- | --- | --- | --- |
|  |  | **2003-5** | **2006-8** | **2009-11** | **2012-14** |
| **MoM** | 18,339 | 2,134 (11.6%) | 11,140 (60.7%) | 4,973 (27.1%) | 92 (0.5%) |
| **Resurfacing** | 18,974 | 4,611 (24.3%) | 9,071 (47.8%) | 4,226 (22.3%) | 1,066 (5.6%) |
| **Other** | 399,353 | 44,145 (11.1%) | 87,146 (21.8%) | 122,198 (30.6%) | 145,864 (36.5%) |
| **Total** | 436,666 | 50,890 (11.7%) | 107,357 (24.6%) | 131,397 (30.1%) | 147,022 (33.7%) |
