# Supplementary material for: The risk of developing cancer following metal-on-metal hip replacement compared with non metal-on-metal hip bearings: Findings from a prospective national registry “The National Joint Registry of England, Wales, Northern Ireland and the Isle of Man”
Source: PLoS One. 2018 Sep 20;13(9):e0204356. doi: 10.1371/journal.pone.0204356 (PMC6147563; doi:10.1371/journal.pone.0204356)
Supplement: S4 Table — (DOCX) [file pone.0204356.s004.docx]

### S4 Table. Proportion of patients with a record of a previous cancer diagnosis recorded in HES prior to primary hip procedure by bearing type.

| **Bearing type for first primary hip** | **Proportion with previous cancer**  **documented in HES** | **Age at primary (years)**  **Median (IQR)** | **Percentage**  **of males** |
| --- | --- | --- | --- |
| **MoM** | 987/18,339 (5.4%) | 64 (58-71) | 50.7% |
| **Resurfacing** | 409/18,974 (2.2%) | 55 (49-60) | 70.3% |
| **Other** | 31,387/399,353 (7.9%) | 70 (63-77) | 38.6% |
| **Total** | 32,783/436,666 (7.5%) | 70 (62-76) | 40.5% |
