# Supplementary material for: The risk of developing cancer following metal-on-metal hip replacement compared with non metal-on-metal hip bearings: Findings from a prospective national registry “The National Joint Registry of England, Wales, Northern Ireland and the Isle of Man”
Source: PLoS One. 2018 Sep 20;13(9):e0204356. doi: 10.1371/journal.pone.0204356 (PMC6147563; doi:10.1371/journal.pone.0204356)
Supplement: S3 Fig — (DOCX) [file pone.0204356.s007.docx]

S3 Fig. Cumulative risk of a new diagnosis of any type of cancer following primary hip replacement by age at first primary for females.
